# The Antiplatelet Action of S-Nitroso Human Serum Albumin in Whole Blood

**DOI:** 10.3390/biomedicines10030649

**Published:** 2022-03-11

**Authors:** Melina Tsiountsioura, Gerhard Cvirn, Axel Schlagenhauf, Harald Haidl, Kathrin Zischmeier, Nicole Janschitz, Martin Koestenberger, Willibald Wonisch, Margret Paar, Thomas Wagner, Eva-Christine Weiss, Seth Hallström

**Affiliations:** 1 Division of Physiological Chemistry, Otto Loewi Research Center, Medical University of Graz, 8010 Graz, Austria; melina.tsiountsioura@greenbeat.at (M.T.); willibald.wonisch@medunigraz.at (W.W.); margret.paar@medunigraz.at (M.P.); seth.hallstroem@medunigraz.at (S.H.); 2 Division of General Paediatrics, Department of Paediatrics and Adolescent Medicine, Medical University of Graz, 8036 Graz, Austria; axel.schlagenhauf@medunigraz.at (A.S.); harald.haidl@medunigraz.at (H.H.); martin.koestenberger@medunigraz.at (M.K.); 3 Department of Pharmacology and Toxicology, University of Graz, 8010 Graz, Austria; kathrin.zischmeier@edu.uni-graz.at (K.Z.); nicole.janschitz@gmx.at (N.J.); 4 Department of Blood Group Serology and Transfusion Medicine, Medical University of Graz, 8036 Graz, Austria; thomas.wagner@medunigraz.at; 5 Department of Obstetrics and Gynecology, Medical University of Graz, 8036 Graz, Austria; ev.weiss@medunigraz.at; 6 Ludwig Boltzmann Institute for Cardiovascular Research at the Center for Biomedical Research, Medical University of Vienna, 1090 Vienna, Austria

**Keywords:** nitric oxide donors, platelet function, impedance aggregometry

## Abstract

Nitric oxide donors (NO-donors) have been shown to have therapeutic potential (e.g., ischemia/reperfusion injury). However, due to their release rate/antiplatelet properties, they may cause bleeding in patients. We therefore studied the antiplatelet effects of the two different NO-donors, i.e., S-NO-Human Serum Albumin (S-NO-HSA) and Diethylammonium (Z)-1-(*N*,*N*-diethylamino)diazen-1-ium-1,2-diolate (DEA-NONOate) in whole blood (WB) samples. WB samples were spiked with S-NO-HSA or DEA-NONOate (100 µmol/L or 200 µmol/L), and the NO release rate (nitrite/nitrate levels via HPLC) and antiplatelet efficacy (impedance aggregometry, platelet function analyzer, Cone-and-platelet analyzer, thrombelastometry) were assessed. S-NO-HSA had a significantly lower NO release compared to equimolar concentrations of DEA-NONOate. Virtually no antiplatelet action of S-NO-HSA was observed in WB samples, whereas DEA-NONOate significantly attenuated platelet function in WB. Impedance aggregometry measurements revealed that Amplitudes (slope: −0.04022 ± 0.01045 ohm/µmol/L, *p* = 0.008) and Lag times (slope: 0.6389 ± 0.2075 s/µmol/L, *p* = 0.0051) were dose-dependently decreased and prolonged by DEA-NONOate. Closure times (Cone-and-platelet analyzer) were dose-dependently prolonged (slope: 0.3738 ± 0.1403 s/µmol/L, *p* = 0.0174 with collagen/ADP coating; slope: −0.5340 ± 0.1473 s/µmol/L, *p* = 0.0019 with collagen/epinephrine coating) by DEA-NONOate. These results in WB further support the pharmacological potential of S-NO-HSA as an NO-donor due to its ability to presumably prevent bleeding events even at high concentrations up to 200 µmol/L.

## 1. Introduction

The free radical nitric oxide (NO) is a biological signaling molecule regulating a wide range of important cellular functions [1,2,3]. Administration of exogenous NO as inhalation therapy has received considerable attention, mainly due to its therapeutic ability to exert profound hemodynamic effects [4,5]. A systematic review analyzed the biomedical literature to determine the effects of NO-donor agent administration on ischemia/reperfusion (I/R) injury in human subjects. In most of the studies, patients treated with NO-donor agents experienced reduced I/R injury compared with controls [6].

Besides their most prominent function, vasorelaxation, NO-donors have been shown to act as anti-inflammatory and neuroprotective agents and are able to influence cellular oxidative status [7]. Moreover, NO-donors have been found to reduce platelet activation [8,9]. NO inhibits platelets by elevating cyclic GMP [10]. NO-mediated elevation of cGMP results in a marked decrease in the number of fibrinogen molecules bound to the platelet [11,12], to an inhibition of intracellular calcium flux [13,14], and an inhibition of platelet secretion [15].

Particularly, the damping effect of NO-donors on platelet aggregation might shift the (NO-treated) patients’ hemostatic system toward hypocoagulability and, thus, toward bleeding. It has been shown in adults with ARDS (acute respiratory distress syndrome) that inhaled NO (iNO) caused prolonged bleeding times, inhibition of platelet aggregation, and reduced P-selectin expression and fibrinogen binding [16,17]. However, iNO has an extremely short half-life (i.e., few seconds), impairing its clinical use in pulmonary diseases [18]. A higher incidence of intracranial hemorrhages has been shown in NO-treated preterm infants with RDS (respiratory-distress syndrome) [19]. Therefore, administration of NO-donors, particularly of iNO or the common low-molecular-weight NO-donors that rapidly release relatively high amounts of NO, can be associated with a bleeding tendency.

We speculate that administration of donors that release low amounts of NO over a long period of time dampen platelet function to a much lesser degree, and, thus, may not provoke bleeding events in patients. One example of such a donor is S-NO-Human Serum Albumin (S-NO-HSA) by virtue of its long-lasting release of limited amounts of NO. S-NO-HSA at dosages of 0.1–0.2 µmol/kg/h provides vasodilatory activity without a decrease in systemic blood pressure [20]. The underlying mechanism is the prevention of uncoupling of endothelial nitric oxide synthase by NO released from S-NO-HSA [20].

Conventionally, platelet function testing is performed in platelet-rich plasma samples. However, while plasma contains many of the coagulation factors implicated in the coagulation process, WB includes phospholipid-bearing cells that support coagulation. In addition, it is also known that hemoglobin present in erythrocytes can scavenge NO [21]. We, therefore, comparatively evaluated the antiplatelet actions of S-NO-HSA and DEA-NONOate in WB samples by applying: (i) impedance aggregometry, (ii) Platelet function analyzer 200, (iii) Cone and Platelet Analyzer (Impact^®^, Linz, Austria), and (iv) Thrombelastometry. The NO release from the two donors was estimated by measurement of nitrite, as well as nitrate, the end products of NO metabolism. Therefore, the aim of our study was to compare the antiplatelet action of S-NO-HSA with that of the fast-releasing NO-donor DEA-NONOate (Diethylammonium (Z)-1-(*N*,*N*-diethylamino)diazen-1-ium-1,2-diolate). We speculate that administration of S-NO-HSA, due to its presumed limited antiplatelet action, might be associated with a significantly lower risk of bleeding in patients compared to administration of common fast NO-releasing donors.

## 2. Materials and Methods

### 2.1. Reagents

S-NO-HSA was prepared as previously described [20,22]. In brief, HSA was processed to yield a maximal free thiol group at position Cys-34 (SH > 0.8 mol/mol protein). Intermolecular disulfides (mixed disulfides) were disassembled prior to nitrosation. The starting material (20% HSA; Biotest, Vienna, Austria) was reduced by mercaptoethanol (10 to 20-fold molar excess; buffer [mmol/L]: sodium phosphate 1, ethylenediaminetetraacetic acid 2, and sodium chloride 150 adjusted to pH = 6.0–6.2 with hydrochloric acid (HCl); 12 to 48 h at 4 °C under nitrogen) and purified by means of gel-permeation chromatography (TSK-HW40F; mobile phase: H_2_O).

Thiol nitrosation was affected with sodium nitrite at a ratio of 1:1 to 1:1.5 of freely available thiol groups to nitrite in 0.2 mol/L HCl (pH = 1.5–2.5) for 30 min at 25 °C. After neutralization with 1 mol/L sodium hydroxide, S-NO-HSA was purified by gel-permeation chromatography (TSK-HW40F; mobile phase: H_2_O) and lyophilized. HSA (control) was also purified by gel-permeation chromatography (TSK-HW40F; mobile phase: H_2_O) and lyophilized. S-NO-HSA was dissolved in 0.9% sodium chloride solution. Stock solutions containing 1 mmol/L of S-NO-HSA and HSA in 0.9% sodium chloride solution were prepared.

DEA-NONOate was purchased from Cayman Chemicals Co. (Ann Arbor, MI, USA), and a stock solution was prepared containing 1 mmol/L of DEA-NONOate in 0.9% sodium chloride solution.

Recombinant human Tissue Factor (TF) thromboplastin (Innovin^®^) was obtained from Dade Behring Marburg GmbH (Marburg, Germany). The lyophilized product was dissolved in 10 mL of distilled water and subsequently diluted at a ratio of 1:1000 in 0.9% sodium chloride solution (TF-stock solution).

### 2.2. Subjects

A total of 22 healthy men (age 27 to 47 years) were recruited. The study was conducted in accordance with the Declaration of Helsinki and was approved by the local ethics committee (31-279 ex 18/19, 02 July 2020). Exclusion criteria were characterized as medication within the last two weeks, which might influence coagulation, as well as renal or liver disease and coagulation disorders. Subjects’ characteristics were 36.4 ± 6.9 years, 81.8 ± 7.3 kg body weight, 1.8 ± 0.1 m height, and 25.5 ± 3.7 kg/m² body mass index.

### 2.3. Blood Collection and Preparation

Seven mL of blood from the antecubital vein were collected into pre-citrated Vacuette^®^ marked tubes (Greiner Bio-one GmbH, Kremsmünster, Austria) containing 3.8% sodium citrate. WB measurements (impedance aggregometry, platelet function tests, TEM) were performed within 3h of blood sampling. S-NO-HSA, DEA-NONOate, and HSA levels were adjusted to 100 or 200 µmol/L by the addition of respective amounts of the stock solutions (50 µL, 100 µL) to 500 µL WB prior to the measurements. WB samples without any addition except 100 µL physiological sodium chloride were used as controls. One aliquot of the remaining WB was centrifuged (room temperature, 12 min, 150 g) in order to obtain platelet-rich plasma (PRP). Another blood aliquot was centrifuged (room temperature, 15 min, 500 g) in order to obtain (autologous) platelet-poor plasma (PPP). After counting, PRP was diluted with PPP to contain 100,000 platelets/µL. S-NO-HSA, DEA-NONOate, and HSA levels in the diluted PRP samples (1 mL) were adjusted to 10 µmol/L or 20 µmol/L by addition of respective amounts of stock solutions (10 µL, 20 µL) prior to the measurements. PRP samples without any addition were measured as absolute controls. Platelet counts were measured by means of the Sysmex KX-21 N Automated Hematology Analyzer (Sysmex, Illinois, IL, USA).

### 2.4. Sampling

The blood (7 mL) was drawn from the antecubital vein from the seated subject into pre-citrated Vacuette^®^ marked tubes (Greiner Bio-one GmbH, Kremsmünster, Austria) containing 3.8% sodium citrate. WB measurements (impedance aggregometry, platelet function tests, TEM) were performed within 3h of blood sampling. One aliquot of the remaining WB was centrifuged (room temperature, 12 min, 150 g) in order to obtain PRP. Another aliquot was centrifuged (room temperature, 15 min, 500 g) in order to obtain (autologous) PPP. PRP was prepared to contain 100 000 platelets/µL by addition of appropriate volumes of PPP. S-NO-HSA, DEA-NONOate, and HSA levels were raised to 10 µmol/L or 20 µmol/L by addition of the respective amounts of stock solutions (10 µL, 20 µL) to 1 mL of PRP. Platelet counts were measured by means of the Sysmex KX-21 N Automated Hematology Analyzer (Sysmex, IL, USA).

### 2.5. Analysis of Nitrite and Nitrate

Five hundred μL aliquots (WB or PRP) were taken immediately after impedance aggregation measurements and centrifuged at room temperature for 15 min at 500× *g*. Subsequently, 200 µL of the supernatant (diluted plasma) were ultrafiltered (mol weight cut off: 10 kDa) by centrifugation at 4 °C for 20 min at 2000 *g* using Microcon^®^-10 Centrifugal filters from Merck Chemicals and Life Sciences GmbH (Vienna, Austria). In addition, some WB and PRP samples were processed immediately after the addition of the NO-donors. Subsequently, the sample ultrafiltrates (100 μL) were diluted with double-distilled water (1:2, v/v). Determination of nitrate in the diluted sample ultrafiltrates was performed in principle according to a previously published method [23] with some modifications. In brief, HPLC consisted of an L-2200 autosampler, two L-2130 HTA pumps, and an L-2450 diode array detector (all: VWR Hitachi, VWR, Vienna, Austria). Separation was performed on a Hypersil ODS (5 µmol/L; 250 × 4 mm I.D.) with 10.0 min isocratic elution (buffer A: 0.1 mol/L NaH_2_PO_4_, pH = 5.5, containing 5.9 mmol/L tetrabutylammonium hydrogen sulphate), followed by a linear gradient to 20% buffer B (buffer B: 0.1 mol/L NaH_2_PO_4_, pH = 5.5, containing 5.9 mmol/L tetrabutylammonium hydrogen sulphate/acetonitrile −75/25%; *vol/ vol*) within another 10 min. The detector signals (absorbance at 205 nm) were recorded, and the program EZchrom Elite (VWR) was used for data acquisition and analysis.

The injection volume of samples and standard solutions was 40 µL. Retention time for nitrite was ~7.80 min for nitrate and ~14.5 min for nitrate. The simultaneous determination of nitrite and nitrate in the samples utilizing this method was impossible due to an interfering substance eluting with nitrite. We therefore analyzed nitrite by a second method, as described below. Nitrite was determined in principle according to a previously described fluorometric HPLC method [24] utilizing the reaction of nitrite with 2,3-diaminonaphthalene (DAN; obtained from Sigma-Aldrich, Vienna, Austria).

In brief, 25 µL diluted sample ultrafiltrate was further diluted with 75 µL 0.9% NaCl, and the so-produced 100 µL sample was incubated at 24°C with 10 µL of 316 µmol/L DAN (in 0.62 mol/L HCl) for 10 min, followed by the addition of 10 µL of 2.8 mol/L NaOH. This reaction mixture was directly used for chromatographic separation (injection volume: 5–20 µL) of the formed 2,3-naphthotriazole (NAT). Nitrite standards (range: 0–2 µmol/L) were derivatized accordingly. Sodium phosphate, sodium hydroxide, and sodium nitrite were purchased from Roth (Karlsruhe, Germany). Our HPLC separation conditions have been previously reported [25]. The sum of nitrite and nitrate was calculated from the obtained results.

### 2.6. Impedance Aggregation Assay

Platelet aggregation in both WB and PRP samples was performed using a Chrono-Log Aggregometer Model 590 from Probe and Go (Endingen, Germany), which is based on the impedance method [26]. WB or PRP samples were incubated with increasing concentrations of S-NO-HSA or DEA-NONOate for 2 min at 37 °C prior to measurement. Impedance aggregometry results are expressed as amplitude (or maximum aggregation) in ohm at six minutes after reagent addition and as lag time (or aggregation time) in seconds, the time interval until the onset of platelet aggregation. The rate of platelet aggregation is expressed as the slope in ohm/min. Collagen (2 µg/mL final concentration), purchased from Probe and Go (Endingen, Germany), was used as a platelet agonist, as previously described [27,28].

### 2.7. Platelet Function Analyzer 200

Using the PFA 200 from Siemens Healthcare Diagnostics (Vienna, Austria), primary hemostasis is simulated with an in vitro quantitative measurement of platelet adhesion and aggregation in WB. The system uses citrated WB (800 µL, spiked with increasing concentrations of S-NO-HSA or DEA-NONOate) that is aspired under high shear stress rates through an aperture cut into a membrane coated with collagen (a subendothelial protein generally believed to be the initial matrix for platelet attachment) and either ADP or epinephrine. In response to the local shear stress and the agonists in the membrane, platelets are activated, adhere to collagen in the membrane surrounding the aperture, and aggregate until a stable platelet plug occludes the blood flow through the aperture. This time period recorded by the instrument is designated as the closure time (CloT), representing a measure of platelet-dependent hemostasis, in particular platelet activation, adherence, and aggregability [29].

### 2.8. Whole Blood Platelet Adhesion/Aggregation Assay

Platelet adhesion and aggregation were assessed using a Cone and Platelet Analyzer (CPA) (DiaMed, Linz, Austria) as described previously [30]. Briefly, 130 µL of citrated WB (spiked with S-NO-HSA or DEA-NONOate) was placed in polystyrene tubes and allowed to flow (1300 s^−1^) for two minutes using a rotating Teflon cone. Subsequently, the wells were washed with PBS, stained with May-Grünwald solution, and analyzed with an image analysis system. Surface coverage (SC) and average size (AS) were determined to elucidate platelet function. SC, representing platelet adhesion, is expressed as the percentage of total area covered by platelets. AS, representing platelet aggregation, is defined as the average size of the surface-bound objects.

### 2.9. Whole Blood Tissue Factor-Triggered TEM Assay

The clot formation process was monitored using the TEM coagulation analyzer (ROTEM^®^05) from Matel Medizintechnik (Graz, Austria). The period of time from adding the trigger to initial fibrin formation is designated as the “Coagulation time” (CT); the time until the amplitude reaches 20 mm refers to the “Clot formation time” (CFT). “Maximum clot firmness” (MCF) reflects clot stability, and the “alpha angle” indicates the velocity of fibrin built-up and cross-linking. The final sample volume was 340 µL. Clot formation was initiated by the addition of 40 µL of “trigger solution” (containing 0.35 pmol/L TF and 3 mmol/L CaCl_2_, final concentration) to 300 µL of citrated WB (spiked with increasing concentrations of S-NO-HSA or DEA-NONOate). This method has been described in detail previously by Sorensen et al. [31]. 

### 2.10. Statistics

The GraphPad 8.0 Prism package was used for statistical evaluation. ANOVA and Bonferroni post-test were used to evaluate differences in plasma levels of nitrite/nitrate. The Mann–Whitney test was used to compare nitrite levels (pre vs. post aggregation) in WB samples. Linear regression was used for statistical evaluation of the effects of increasing concentrations of S-NO-HSA and DEA-NONOate on coagulation parameters. All *p*-values of ≤0.05 were considered statistically significant. * *p* ≤ 0.05, ** *p* ≤ 0.01, *** *p* ≤ 0.001.

## 3. Results

### 3.1. Nitrite/Nitrate Amounts in the Presence of Increasing Concentrations of NO-Donors in WB

**Figure 1:**
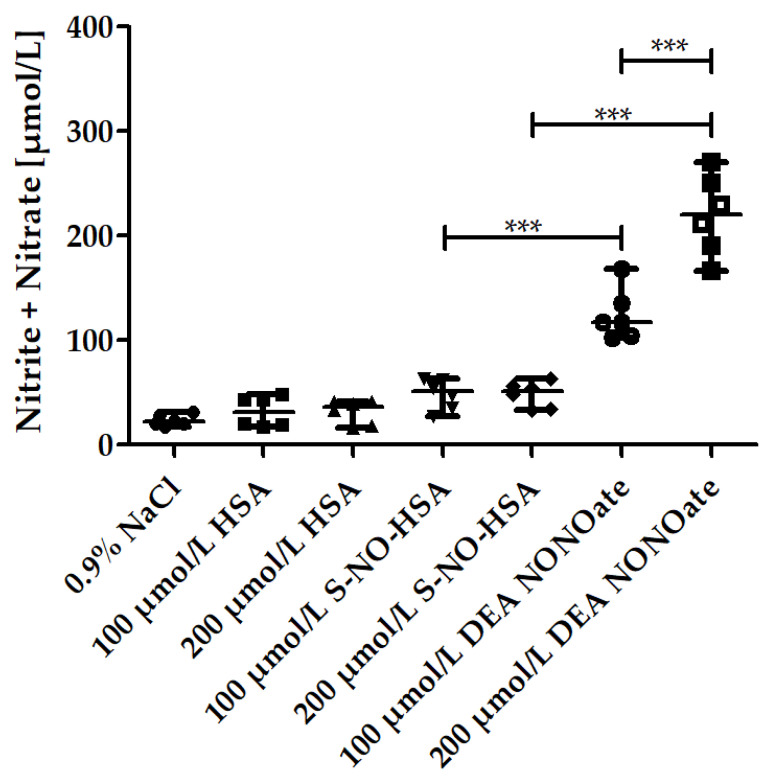
Nitrite/nitrate amounts in the presence of increasing concentrations of S-NO-HSA or DEA-NONOate in WB. The increase in the sum of nitrite and nitrate amounts in µmol/L served as a measure for NO release from the NO-donors in WB. Significantly higher amounts of NO were released by DEA-NONOate (100 and 200 µmol/L final concentration) than by equimolar amounts of S-NO-HAS after 6 min aggregation. ANOVA and Bonferroni post-test were used to evaluate differences in plasma levels of nitrite/nitrate. The results are presented as median with range (*n* = 6). *** *p* ≤ 0.001.

WB samples (*n* = 6) were spiked to contain 100 µmol/L or 200 µmol/L of either S-NO-HSA or DEA-NONOate. Sodium chloride solution or HSA served as controls, respectively. The associated increase in the sum of nitrite and nitrate concentrations reflects the NO release from the donors. Addition of sodium chloride solution or HSA had no influence on the sum of nitrite and nitrate, which remained within the normal physiological range [32], as shown in Figure 1. The NO release from S-NO-HSA was significantly lower than that from equimolar concentrations of DEA-NONOate. For example, the sum of nitrite and nitrate median amounts increased from 21.89 µmol/L (physiological sodium chloride) to 51.19 µmol/L in the presence of 200 µmol/L S-NO-HSA but to 219.9 µmol/L in the presence of 200 µmol/L DEA-NONOate in WB (*p* < 0.0001, Figure 1). In WB, the read out was the sum of nitrite and nitrite, as due to the presence of oxygen (erythrocytes). The initially formed nitrite is further converted to nitrate. This conversion was also determined by measuring the nitrite values at the concentration of 200 µmol/L of the NO-donors 2 min after addition of the compounds (start point of measurement in the adhesion/aggregation assay) and after 6 min aggregation (endpoint of measurement). The nitrite median values with 200 µmol/L S-NO-HSA dropped slightly but not significantly from 3.37 to 2.29 µmol/L (*n* = 6, *p* = 0.1797). In the presence of 200 µmol/L DEA-NONOate the nitrite median amounts dropped significantly from 64.74 to 26.56 µmol/L (*n* = 6, *p* = 0.0022) within the 6 min aggregation measurement period.

### 3.2. Effects of Increasing Concentrations of NO-Donors on Impedance Aggregometry Values in WB

**Figure 2:**
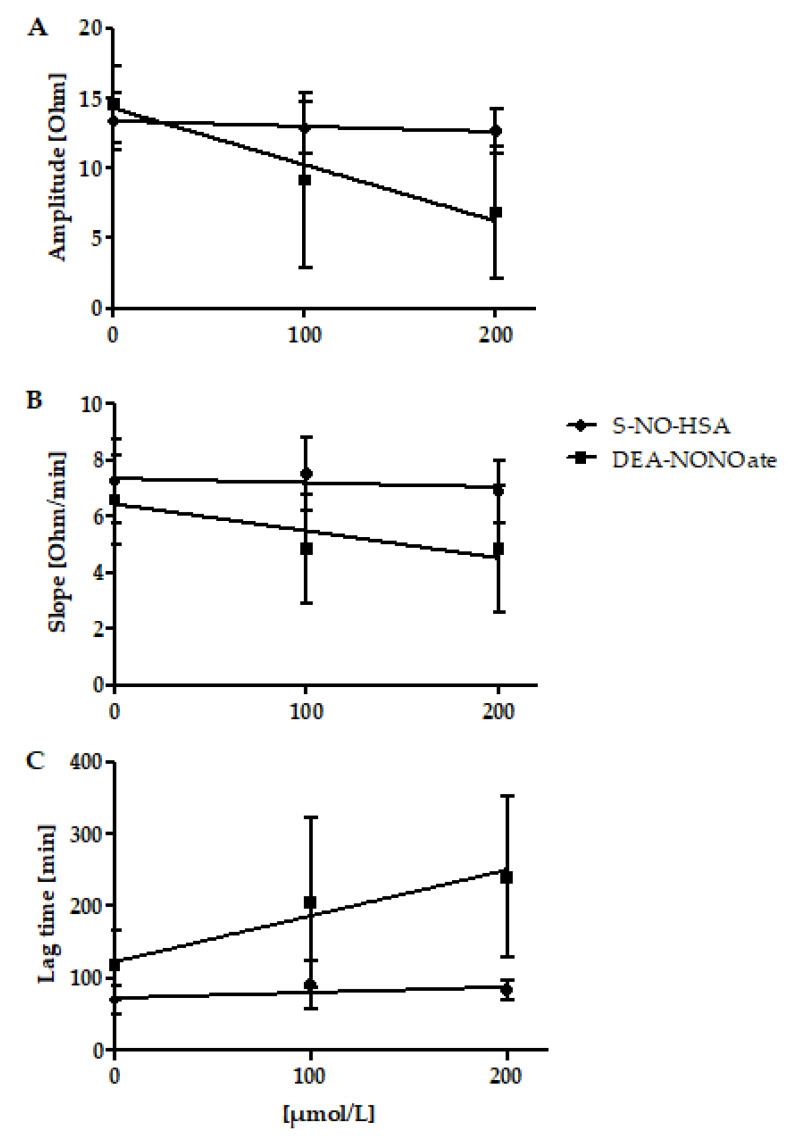
Effects of increasing concentrations of S-NO-HSA or DEA-NONOate on impedance aggregometry values in WB. Panel (**A**): Addition of S-NO-HSA had no effect on amplitudes, while the addition of equimolar amounts of DEA-NONOate led to decreased amplitudes. Linear regression analysis showed that slopes differed significantly (p = 0.0009. Panel (**B**): Addition of S-NO-HSA had no influence on Slopes while the addition of equimolar amounts of DEA-NONOate caused a dose-dependent decrease of Slopes. Linear regression analysis showed that slopes differ significantly (*p* = 0.0009). Panel (**C**): Addition of S-NO-HSA had no effect on Lag times, while the addition of equimolar amounts of DEA-NONOate led to prolonged lag times. Linear regression analysis showed that the slopes differ significantly (*p* = 0.0061). The results are presented as mean ± SD; *n* = 16 (S-NO-HSA), *n* = 13 (DEA-NONOate). The data at 0 µmol/L represent the values of the appropriate controls (no NO-donor added).

Addition of S-NO-HSA had no significant effect on platelet aggregation values in WB samples (*n* = 16). Amplitudes (slope: −0.003864 ± 0.004019 ohm/µmol/L, *p* = 0,3440), Slopes (slope: −0.001477 ± 0.002886 ohm/µmol/Lmin, *p* = 0.6125), and lag times (slope: 0.07654 ± 0.04850 s/µmol/L, *p* = 0.1257) were not significantly altered in the presence of increasing NO-donor concentrations, Figure 2, panels A, B, and C. In contrast, significant antiaggregatory effects of DEA-NONOate were observed in WB samples (*n* = 13). Amplitudes (slope: −0.04022 ± 0.01045 ohm/µmol/L, *p* = 0.008), Slopes (slope: −0.009545 ± 0.004513 ohm/µmol/L·min, *p* = 0.0460), and Lag times (slope: 0.6389 ± 0.2075 s/µmol/L, *p* = 0.0051) were dose-dependently decreased and prolonged in the presence of increasing concentrations of DEA-NONOate (Figure 2, panels A, B, and C).

### 3.3. Effects of Increasing Concentrations of NO-Donors on PFA 200 Values in WB

**Figure 3:**
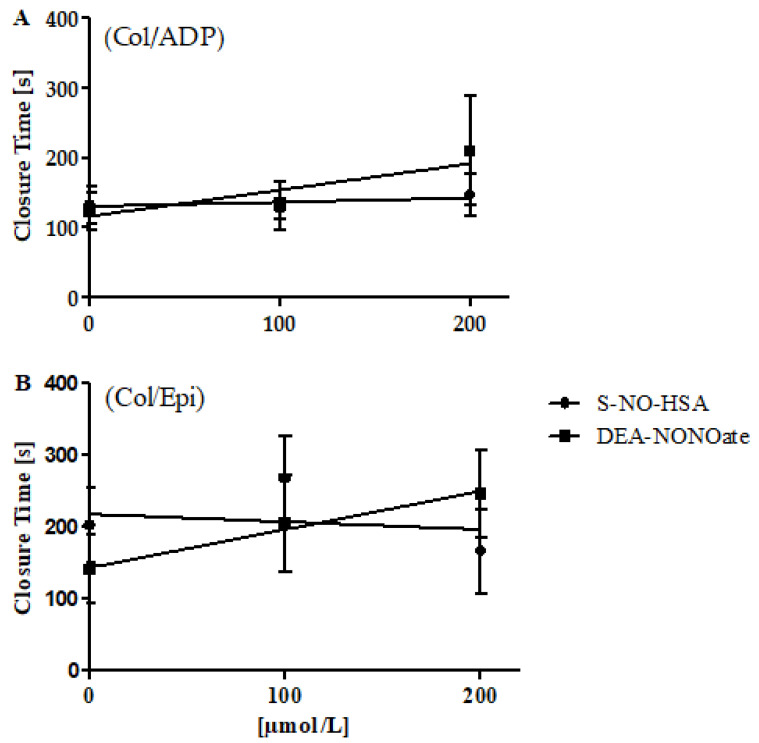
Effects of increasing concentrations of S-NO-HSA or DEA-NONOate on PFA 200 values in WB. Panel (**A**): Utilizing cartridges coated with collagen/ADP, addition of S-NO-HSA (*n* = 12) had no effect on CloTs, while the addition of equimolar concentrations of DEA-NONOate (*n* = 8) led to dose-dependently prolonged CloTs. Linear regression analysis showed that slopes differ significantly (*p* = 0.02639). Panel (**B**): Utilizing cartridges coated with collagen/epinephrine, addition of S-NO-HSA (*n* = 12) had no effect on CloTs, while the addition of equimolar amounts of DEA-NONOate (*n* = 10) led to prolonged CloTs. Linear regression analysis showed that the slopes differ significantly (*p* = 0.006435). The results are presented as mean ± SD. The data at 0 µmol/L represent the values of the appropriate controls (no NO-donor added).

The addition of S-NO-HSA had no significant antiplatelet effect in WB samples (*n* = 12). CloT was not significantly altered (slope: 0.05939 ± 0.06422 s/µmol/L, *p* = 0.3651, for cartridges coated with collagen/ADP; slope: −0.1059 ± 0.1616 s/µmol/L, *p* = 0.5191, for cartridges coated with collagen/epinephrine) by increasing concentrations of S-NO-HSA, as shown in Figure 3 (panels A and B). In contrast, DEA-NONOate exerted significant antiplatelet action in WB samples (*n* = 8). CloT was dose-dependently prolonged (slope: 0.3738 ± 0.1403 s/µmol/L, *p* = 0.0174 for cartridges coated with collagen/ADP; slope: −0.5340 ± 0.1473 s/µmol/L, *p* = 0.0019 for cartridges coated with collagen/epinephrine) in the presence of increasing concentrations of DEA-NONOate (Figure 3, panel A and B).

### 3.4. Effects of Increasing Concentrations of NO-Donors on CPA Values in WB

The addition of S-NO-HSA had no significant effects on CPA values in WB samples (*n* = 19). SC (slope: −0.01594 ± 0.008340%/µmol/L, *p* = 0.0640) and AS (slope: −0.0414 ± 0.02130 µm²/µmol/L, *p* = 0.0620) were not significantly altered by increasing concentrations of S-NO-HSA. Similarly, the addition of DEA-NONOate had no significant effect on CPA values in WB samples (*n* = 17). SC (slope: 0.01046 ± 0.01120%/µmol/L, *p* = 0.3575) and AS (slope: −0.1019 ± 0.05909 µm²/µmol/L, *p* = 0.0944) were not altered by increasing the concentrations of DEA-NONOate.

### 3.5. Effects of Increasing Concentrations of NO-Donors on TEM Values in WB

The addition of S-NO-HSA had no significant effect on CT (slope: 0.1425 ± 0.1092 s/µmol/L, *p* = 0.2026), CFT (slope: 0.3269 ± 0.1772 s/µmol/L, *p* = 0.0757), and alpha (slope: −0.0477 ± 0.02120°/µmol/L, *p* = 0.0647) in WB samples (*n* =15). However, MCF dose-dependently decreased in the presence of increasing concentrations of S-NO-HSA (slope: −0.02188 ± 0.01042 mm/µmol/L, *p* = 0.0449). The addition of DEA-NONOate had no significant effect on all TEM values in WB samples (*n* = 17). CT (slope: −0.1252 ± 0.08235 s/µmol/L, *p* = 0.1353), CFT (−0.1653 ± 0.08895 s/µmol/Lol/L, *p* = 0.0697), MCF (slope: 0.009038 ± 0.006832 mm/µmol/L, *p* = 0.1924), and alpha (slope: 0.02779 ± 0.01444 °/µmol/L, *p* = 0.0605) were not affected by DEA-NONOate addition.

### 3.6. Nitrite/Nitrate Levels in the Presence of Increasing Concentrations of NO-donors in PRP

**Figure 4:**
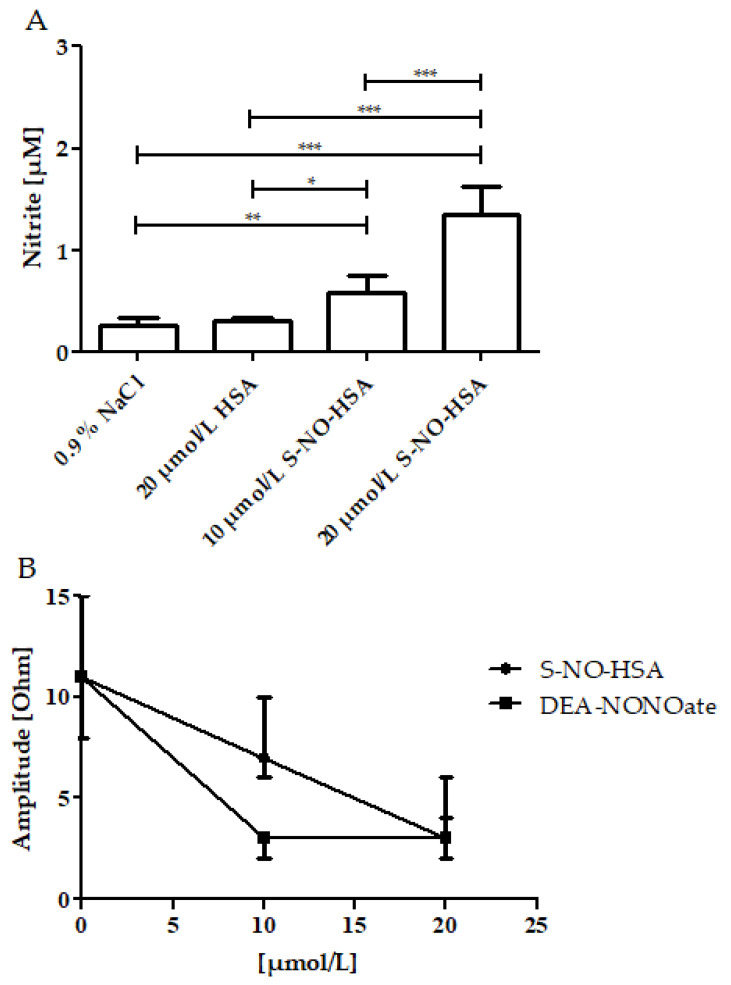
Effects of increasing concentrations of S-NO-HSA or DEA-NONOate on nitrite levels and on impedance aggregometry amplitudes in PRP. Panel (**A**): Nitrite levels dose-dependently increased in the presence of increasing concentrations of S-NO-HSA (*n* = 6). At 20 µmol/L DEA-NONOate, nitrite levels were 21.59 (16.51–32.75) µmol/L (*p* < 0.001 vs. physiological sodium chloride; data not illustrated in the figure). ANOVA and Bonferroni post-test were used to evaluate differences in plasma nitrite levels. The results are presented as median with range. * *p* ≤ 0.05; ** *p* ≤ 0.01; *** *p* ≤ 0.001. Panel (**B**): Both S-NO-HSA (*n* = 17) and DEA-NONOate (*n* = 8) efficiently inhibited platelet aggregation. Virtually no platelet aggregation occurred in the presence of 20 µmol/L S-NO-HSA and 10 µmol/L DEA-NONOate (*n* = 8), respectively. The results are presented as mean ± SD. The data at 0 µmol/L represent the values of the appropriate controls (no NO-donor added).

PRP samples (*n* = 6) were spiked to contain 10 µmol/L or 20 µmol/L of either S-NO-HSA or DEA-NONOate. Physiological sodium chloride solution or HSA served as controls. In PRP, the associated increase of nitrite concentrations reflects the NO release from the donors. The addition of HSA had no significant influence on nitrite levels when compared to physiological sodium chloride, as shown in Figure 4 (panel A). The nitrite median levels significantly increased in the presence of 10 µmol/L of S-NO-HSA (0.585 µmol/L, *p* < 0.05), as well as in the presence of 20 µmol/L S-NO-HSA (1.350 µmol/L, *p* < 0.001) as compared to physiological sodium chloride. Comparable to the WB measurements, markedly higher amounts of NO were released from DEA-NONOate. In the presence of 20 µmol/L DEA-NONOate, nitrite levels were 21.59 (16.51–32.75) µmol/L (*p* < 0.001 vs. physiological sodium chloride). There was no significant difference concerning PRP nitrate levels between HSA, S-NO-HSA and DEA-NONOate at all tested concentrations.

### 3.7. Effects of Increasing Concentrations of NO-Donors on Impedance Aggregometry Values in PRP

Both S-NO-HSA (*n* = 17) and DEA-NONOate (*n* = 8) exerted efficient antiaggregatory effects in PRP samples. S-NO-HSA at concentrations of 20 µmol/L and DEA-NONOate at both tested concentrations of 10 µmol/L and 20 µmol/L completely abolished platelet aggregation (Figure 4, panel B).

## 4. Discussion

The experiments presented here demonstrate that the NO-donor S-NO-HSA (at concentrations up to 200 µmol/L) exerts virtually no antiplatelet actions in WB samples, whereas equimolar concentrations of the NO-donor DEA-NONOate caused significant inhibition of platelet function.

This difference is apparently attributable to the different modes by which NO is being released from the donors. S-NO-HSA has been shown to be a NO-donor releasing low amounts of NO over a prolonged period of time [22,32] whereas DEA-NONOate is a potent and fast-releasing NO donor [33].

However, to our knowledge, no study has been conducted to evaluate the antiplatelet action of low-molecular-weight and high-molecular-weight NO-donors in WB. This was the aim of our study.

The release of NO was quantified in our study by measuring the respective increases of nitrite and nitrate plasma levels by HPLC methods. Depending on the environment, nitrite can further be oxidized to nitrate (WB). Nitrate is a stable metabolite of NO. Both nitrite and nitrate are accessible for quantitative analysis [34]. Our experiments show, as expected, that the high-molecular-weight NO-donor S-NO-HSA releases significantly lower amounts of NO than equimolar concentrations of the low-molecular-weight NO-donor DEA-NONOate. In WB, mainly the increase of nitrate and to a much lesser extent nitrite reflected the NO release of the compounds. For the quantification, the sum of nitrite and nitrate was used in WB. The data are in good agreement with the findings of Scorza et al., who showed that the release of NO from low-molecular-weight donors is faster and quantitatively higher than that from high-molecular-weight donors [35]. Consistently, it has been shown that S-NO-HSA has a prolonged half-life in comparison to low-molecular-weight S-nitroso thiols [22]. DEA-NONOate as a low-molecular-weight NO releasing compound was chosen for comparison as it is a very fast NO releasing low-molecular-weight NO-donor according to literature [36,37] and our own observation.

In accordance with our findings, significantly lower antiplatelet actions of high- compared with low-molecular-weight NO-donors were reported in a previously published paper. The exposure of platelets to low-molecular-weight donors inhibited platelet aggregation by >95%, while the high-molecular-weight donor S-nitrosoalbumin was much less effective [38]. It should be mentioned that S-nitrosoalbumin in their study was generated in a different way in comparison with the methods and standards applied in our study. The experiments were performed in a purified system using washed platelets. However, our study confirms these findings in the physiologically more relevant system of WB.

A previous study also found significantly lower antiplatelet actions of high- compared with low-molecular-weight NO donors. The exposure of platelets to low-molecular-weight donors inhibited platelet aggregation by >95%, while the high-molecular-weight donor S-nitrosoalbumin was much less effective [38]. It has to be mentioned that S-nitrosoalbumin in this study has not been produced in accordance with the methods and standards applied in our study. The experiments in the mentioned study were performed in a purified system using washed platelets. However, our study confirms these findings in the physiologically more relevant system of WB.

Interestingly, our experiments demonstrate that both S-NO-HSA and DEA-NONOate exerted significant antiplatelet actions in PRP samples at the tested concentrations of 10 µmol/L and 20 µmol/L. That S-NO-HSA reveals an effect in PRP at these concentrations is apparently attributable to the absence of erythrocytes (hemoglobin) in PRP samples. Hemoglobin present in erythrocytes is known to be the physiological scavenger of NO in vivo [35,39]. In the presence of hemoglobin (in WB samples), DEA-NONOate alone is capable of releasing the required amounts of NO to dampen platelet function, whereas in the absence of hemoglobin (in PRP samples), both donors are capable of dampening platelet function. Correspondingly, in WB samples, approximately 100 µmol/L of DEA-NONOate were required for a ~40% reduction of platelet aggregation (amplitude), whereas in PRP samples, the tested concentration of 10 µmol/L of DEA-NONOate almost completely abolished platelet aggregation.

It has to be stated that the amount of NO being released per unit of time is not the only criterion rendering a NO donor an efficient platelet antagonist [38,40]. It has been suggested that NO-donors must interact directly with platelets to cause physiological responses [38,41]. Shah et al. have shown that transfer of nitroso groups from NO donors onto exofacial thiols on the platelet surface conveys their antiplatelet action [38]. We, therefore, conclude that the limited antiplatelet action of S-NO-HSA observed in our experiments is attributable to the low amounts of NO being released as well as therefore a less transmission of NO onto the platelet surface.

The findings presented herein have clinical implications. Reperfusion (re-establishing blood flow to ischemic organs and tissues) is an essential step in many surgical procedures [22,42,43]. However, reperfusion can be associated with changes in vasomotility and increased microvascular permeability, causing massive edema formation and tissue destruction [44,45,46]. Exogenous NO donors have beneficial effects in the reduction of I/R injury by preserving the function of endothelial nitric oxide synthase, thereby stabilizing the basal production of NO and decreasing the production of oxidized species [47,48].

However, besides these positive and desired effects, administration of NO donors, due to their antiplatelet properties, could induce a bleeding tendency in treated patients [16,17,49,50]. The findings of our study suggest that S-NO-HAS in particular might be an NO donor afflicted with low bleeding risk. We show herein that S-NO-HSA exerts virtually no antiplatelet effects in WB up to concentrations of 200 µmol/L. To date, S-NO-HSA is not an approved drug for use in humans. However, preclinical studies using numerous and different animal models suggest that infusion of S-NO-HSA can minimize/prevent I/R-injury [20]. In an I/R model of skeletal muscle in the rabbit, it could be shown that S-NO-HSA infusion at a dose of 0.1 µmol/kg/h leading to NO concentrations in the nanomolar range (<250 nmol/L; measured with a porphyrinic microsensor in vivo) is a powerful tool to prevent or reduce I/R-induced microvessel constriction and muscle perfusion edema [22]. The underlying mechanism is a prevention of uncoupling of endothelial nitric oxide synthesis and thereby prevention of detrimental excess radical formation via peroxynitrite. S-NO-HSA application, e.g., has also been shown to have beneficial effects in wound healing [51], pulmonary hypertension [52], and an LPS model of septic shock in preclinical studies at doses up to 0.5 µmol/kg/h. These doses/concentrations do not affect blood pressure [53]. The application of S-NO-HSA in these concentration ranges (and up to 200 µmol/L) is, according to the present study, not associated with antiplatelet action and, therefore, will not induce a bleeding tendency in treated patients.

However, since the present study is an in vitro study performed in blood samples from healthy donors, future studies in humans upon a possible drug approval are required to further investigate the physiological effects of S-NO-HSA infusion, e.g., the antiplatelet action. In addition, it has to be mentioned that S-NO-HSA produced from our group has until now only been studied in preclinical situations.

## 5. Conclusions

S-NO-HSA even when applied at higher concentrations than required for I/R-injury (e.g., reduction of blood pressure) seems to be a suitable candidate for NO-donor treatment without shifting the patients’ hemostatic system toward bleeding.

## Data Availability

The authors hereby declare that the data presented in this study will be presented upon request by the corresponding author.
